# Ethyl Acetate Extract of *Cynanchi Auriculati Radix* Inhibits LPS-Induced M1 Polarization of RAW264.7 Macrophages and Prolongs the Lifespan of *Caenorhabditis elegans* by Regulating NF-κB and PMK-1/SKN-1 Signaling Pathways

**DOI:** 10.3390/cimb47110934

**Published:** 2025-11-10

**Authors:** Jiawei Fan, Ya Su, Yi Xing, Kun Hu, Jie Ren, Jia Yang

**Affiliations:** 1 School of Pharmacy, Changzhou University, 21 Gehu Road, Changzhou 213164, China; 2 Purchasing and Supply Department, The Affiliated Changzhou No. 2 People’s Hospital of Nanjing Medical University, NO. 68. Gehu Middle Road, Changzhou 213164, China

**Keywords:** *Cynanchum auriculatum* Royle ex Wight., inflammation, aging, NF-κB, p38 MAPK, SKN-1, *Caenorhabditis elegans*

## Abstract

Extracts of *Cynanchi Auriculati Radix* (RCA), derived from the roots of *Cynanchum auriculatum* Royle ex Wight. (CA), have been documented to possess anti-inflammatory and antioxidant properties. However, the molecular mechanisms of their anti-aging action remain unclear. The present study aimed to explore the potential anti-aging components and mechanisms of RCA. LC-MS/MS and network pharmacology were used to identify components and targets. In vitro, LPS-induced RAW264.7 macrophages were used to assess anti-inflammatory effects. In vivo, *Caenorhabditis elegans* models were employed to evaluate lifespan and stress resistance. Five bioactive components were identified. The ethyl acetate extract of RCA (RCAEA) inhibited LPS-induced M1 macrophage polarization by suppressing the expression of NO, PGE2, IL-1β, iNOS, COX-2, TNF-α, and IL-6 via the NF-κB pathway. In *C. elegans*, RCAEA extended lifespan and enhanced oxidative and heat stress resistance, without affecting reproduction. These benefits were mediated by the PMK-1/SKN-1 pathway, as confirmed using mutant strains. RCAEA is a promising anti-aging and anti-inflammatory agent, acting through NF-κB and PMK-1/SKN-1 signaling pathways.

## 1. Introduction

Aging is an unstoppable natural process in which tissues and organs slowly lose their physiological functions. It often leads to related diseases, such as neurodegenerative diseases, immune dysfunction, and cancers, which have a profound impact on human health worldwide [1]. Aging is primarily driven by external stressors, and senescent cells enter a non-dividing state triggered by many factors, including deoxyribonucleic acid (DNA) damage [2], oxidative stress, and chromatin structure changes, leading to accelerated organ and cell degeneration [3]. Inflammation, a chronic and progressive state that accompanies the aging process, is characterized by chronically elevated cytokine levels [4]. At present, anti-inflammatory therapies have been shown to alleviate aging-related diseases and extend lifespan [5]. Therefore, identifying new therapeutic targets and interventions to reduce various aging-related diseases caused by inflammation is important.

Currently, many extracts from natural plants have been demonstrated to possess anti-aging effects [6,7]. To systematically investigate their underlying therapeutic mechanisms [8], advanced bioinformatics tools like network pharmacology (NP) were utilized in the modern pharmacology area [9]. NP can enhance scientific rigor and application value [10,11], thus facilitating the discovery of novel drug targets and molecular pathways and ultimately clarifying drug actions from the perspective of multi-dimensional biological networks [12].

*Cynanchum auriculatum* Royle ex Wight. (CA), commonly known as “Baishouwu” in China, can be converted into tea and starch for food industry use [13]. To date, 151 pure compounds have been identified in *Cynanchi Auriculati Radix* (RCA) and categorized into C_21_-steroids, acetophenones, flavonoids, alkaloids, and terpenoids. C_21_-steroids demonstrate strong anti-tumor activity [14,15]. Furthermore, acetophenones and crude extracts have exhibited immunomodulatory, anti-inflammatory, and antidepressant properties. Other investigations have also revealed that their chemical compositions have considerable medicinal value [16,17]. In contrast, the anti-aging impact of RCA by regulating inflammation-related factors and signaling pathways remains largely unexplored.

Therefore, we hypothesize that the ethyl acetate extract of RCA (RCAEA), for which its chemical components have been identified by liquid chromatography–tandem mass spectrometry (LC-MS/MS), can exert protective effects by reducing lipopolysaccharide (LPS)-induced inflammatory damage in RAW264.7 macrophages through the regulation of inflammatory mediators and macrophage polarization parameters. Subsequently, we assume that RCAEA can demonstrate anti-aging effects by influencing longevity-related biomarkers of *C. elegans* and that its underlying molecular mechanisms can be elucidated. Collectively, these assumed effects and mechanisms are expected to offer new insights into the protective mechanisms of RCAEA in aging processes.

## 2. Materials and Methods

### 2.1. Materials and Reagents

The plant material was harvested from Binhai County, Jiangsu Province, China, in December 2020 and stored in a shaded location for later use. It was identified as RCA by Prof. Jie Ren from Changzhou University, China. Analytical-grade ethanol, petroleum ether, methylene chloride, ethyl acetate, and n-butanol were purchased from Energy Chemistry (Shanghai, China). Trypsin, Arc-Bis, Trisma Base, and Glycine were purchased from Biosharp (Guangzhou, China). The Methyl Thiazolyl Tetrazolium (MTT) Kit, Griess Kit, and Enzyme-Linked Immunosorbent Assay (ELISA) Kit were purchased from Qiao Yi Biotechnology, Co., Ltd. (Hefei, China). Radio Immunoprecipitation Assay (RIPA) Lysate and a BCA Test Kit were purchased from KGI Biotechnology, Co., Ltd. (Nanjing, China). All antibodies were from Beyotime (Shanghai, China). The Ultra-Pure Total RNA Rapid Extraction Kit was from Genenode (Wuhan, China), and the ECL Western Blotting Substrate was from Proteintech (Chicago, IL, USA). Primers for qPCR were synthesized by GenScript Biotech (Nanjing, China). The nuclear and cytoplasmic protein extraction kit (P0028) was purchased from Beyotime (Shanghai, China).

### 2.2. The Preparation for RCAEA

As reported by Su et al. [18], RCAEA was prepared. The extraction and separation flowchart is shown in Figure S1.

### 2.3. LC-MS/MS Instrumentation and Conditions

LC-MS/MS analysis was conducted on an AB SCIEX Instruments 6600 Triple TOF (Applied Biosystems, Foster City, CA, USA) coupled with Prominence-I LC-2030C 3D HPLC system (Shimadzu, Kyoto, Japan). The specific experimental conditions are detailed in the Supplementary Materials.

### 2.4. Network Aharmacology Analysis

Based on the results of LC-MS/MS, the Traditional Chinese Medicine Systems Pharmacology (TCMSP) platform (https://www.tcmsp-e.com/, accessed on 8 August 2025), the PubChem platform (https://pubchem.ncbi.nlm.nih.gov, accessed on 8 August 2025), and the Swiss Target Prediction databases (https://www.swisstargetprediction.ch/, accessed on 8 August 2025) were chosen to obtain the active components targets of RCAEA. The UniProt database (http://www.uniprot.org, accessed on 8 August 2025) was used to standardize gene symbol names. The human gene targets of aging were identified in the GeneCards database (https://www.genecards.org/, accessed on 8 August 2025), OMIM database (https://www.omim.org/, accessed on 8 August 2025), DisGeNET database (https://www.disgenet.org/, accessed on 8 August 2025), and DrugBank database (https://www.drugbank.ca/, accessed on 8 August 2025). The Venny 2.1.0 platform (https://bioinfogp.cnb.csic.es/tools/venny/, accessed on 8 August 2025) was used to screen the common targets of drugs and diseases. Then, the String database (https://string-db.org, accessed on 8 August 2025) and Cytoscape 3.10.0 software were utilized to select the critical targets. Finally, the enrichment analyses of Gene Ontology (GO) functions and the Kyoto Encyclopedia of Genes and Genomes (KEGG) were performed using the STRING online platform (https://string-db.org, accessed on 8 August 2025), and Cytoscape 3.10.1 software was employed to create a network diagram of drug–target–pathway–disease interactions.

### 2.5. Cell Culture and Viability Assays

RAW264.7 macrophage cells (ZQ0098) were provided by Zhong Qiao Xin Zhou Biotechnology Co., Ltd. (Shanghai, China). LPS (L6529-1 mg) was purchased from Sigma-Aldrich (Shanghai, China). The cells were cultured in Dulbecco’s Modified Eagle Medium (DMEM), which contained 10% FBS, 100 U/mL penicillin, and 100 μg/mL streptomycin at 37 °C in a humidified atmosphere of 5% CO_2_. The toxicity of RCAEA (25, 50, 100, 200, 400, and 800 μg/mL) and LPS (100, 200, 400, and 800 ng/mL) was detected according to the absorbance values at 570 nm by the MTT method to determine the concentration to be used for subsequent experiments [19]. Then, cells were treated with RCAEA (25, 50, and 100 μg/mL) and LPS (200 ng/mL). LPS (200 ng/mL) was used for the induction of M1 polarization for 24 h. Nitric oxide (NO) concentrations were measured using the Griess Kit according to the manufacturer’s instructions [20].

### 2.6. ELISA

RAW264.7 cells were treated with RCAEA (25, 50, and 100 μg/mL) or 200 ng/mL LPS for 24 h. Then, the cell supernatant was collected. We carried out the subsequent operations in accordance with the instructions of the ELISA kits (Jianglai Biotechnology Co., LTD., Shanghai, China) [21]. The details are in the Supplementary Materials.

### 2.7. Real-Time Quantitative PCR (qRT-PCR) Assay of RAW264.7 Cells

RAW264.7 macrophages were treated with RCAEA (25, 50, 100 μg/mL) in the presence or absence of LPS (200 ng/mL). Each treatment condition, including different concentrations of RCAEA and LPS, was added in duplicate to a 24-well plate (100 μL per well). Following treatment, the total RNA was extracted using an Ultra-Pure Total RNA Rapid Extraction Kit. Then, cDNA was synthesized by reverse transcription. The *β-actin* gene was used as an internal control. mRNA expression levels of targeted genes were quantified using the 2^−ΔΔCt^ method. The primer sequences and experimental conditions of genes are listed in Tables S1 and S2.

### 2.8. Western Blotting

The method was carried out according to [22,23]. The following primary antibodies were used: β-actin (1:2000), Lamin B (1:2000), inducible nitric oxide synthase (iNOS) (1:5000), cyclooxygenase-2 (COX-2) (1:5000), nuclear factor kappa-B (NF-κB) (1:2000), IκBα (1:5000), and p-IκBα (1:5000). Bands were visualized using a Tanon 5200 imaging system (Tanon, Shanghai, China). The protein expressions were quantified using ImageJ 1.8.0 software (National Institutes of Health, Bethesda, MD, USA).

### 2.9. Immunofluorescence

RAW264.7 cells were cultured in 12-well plates and subjected to the indicated treatments for 24 h. The details are presented in the Supplementary Materials.

### 2.10. Cultivation and Synchronization of Caenorhabditis Elegans (C. elegans)

N2, Bristol wild-type; EU1, *skn-1(zu67) IV/nT1 [unc-?(n754) let-?] (IV;V)*; KU4, *sek-1 (km 4)X* and KU25, *pmk-1(km25) IV*. These strains were sourced from the Caenorhabditis Genetics Center (CGC).

*C. elegans* were cultured on nematode growth medium (NGM), with *Escherichia coli* (*E. coli*) OP50 [22]. After repeatedly rinsing the worms with 1 mL of M9 buffer, they were placed in sterile 1.5 mL EP tubes. Then, 750 μL of lysis buffer was added, and the samples were vortexed for 5 min and centrifuged at 4000 rpm for 2 min. After removing the supernatant, the pellet was washed by adding 1 mL of M9 buffer for rinsing. This washing and centrifugation step was repeated twice. The collected eggs were then transferred onto fresh NGM and incubated at 20 °C until the progeny reached the L4 stage, completing the synchronization process.

### 2.11. Bacterial Growth Rates

As referenced in [24], the experimental conditions were adjusted. The detailed experimental conditions can be found in the Supplementary Materials.

### 2.12. Locomotor Behavior Assays

The synchronized L4-staged worms were cultured on NGM treated with or without RCAEA (0.25, 0.5, 1, 2, 4, 8, and 16 mg/mL) at 20 °C for 4 days to assess their motility. For each group, at least five worms were randomly selected. The number of head wiggles made by the worms in each group within 30 s was measured, with a head wiggle from one side to the other side counted as once [25].

### 2.13. Lifespan Assay

At least fifty synchronized L4-staged worms were randomly selected from each group and cultured on NGM treated with or without RCAEA (1, 2, and 4 mg/mL) at 20 °C. The daily survival of the worms was monitored by transferring surviving worms to fresh NGM until all died. Death was confirmed by the lack of response of the worms to platinum wire stimulation at both the head and tail. Survival rates were calculated, and survival curves were generated [26].

### 2.14. Reproduction Assay

At least five *C. elegans* were cultured as described in Section 2.13. Subsequently, the number of eggs produced was recorded every 24 h for 5 consecutive days [27].

### 2.15. Stress Assay

At least ten *C. elegans* were cultured as described in Section 2.13 for 48 h.

Oxidative Stress: Worms were transferred to NGM containing 50 μM juglone. The number of surviving worms was counted every 0.5 h until all died. Worms were considered dead when they did not react to a light touch. Survival rates were calculated, and survival curves were generated.

Heat Stress: At least fifteen worms were randomly selected and placed onto a new NGM in a constant-temperature incubator at 35 °C. The number of dead worms, surviving worms, and total worms was counted every 1 h. Survival rates were calculated, and survival curves were generated.

### 2.16. Measurement of Reactive Oxygen Species (ROS)

*C. elegans* were cultured as described in Section 2.13 for 3 days. These worms were exposed to 50 mM H_2_O_2_ for 4 h [28]. The specific experimental protocol is presented in the Supplementary Materials.

### 2.17. Lipofuscin Assays

*C. elegans* were cultured as described in Section 2.13 for 12 days. After spawning, at least five worms from each group were randomly selected and anesthetized with 0.1% NaN_3_. The spontaneous fluorescence of lipofuscin in the worms was observed and photographed at 100× magnification [29]. The lipofuscin accumulation level was quantified based on fluorescence intensity using ImageJ software.

### 2.18. qRT-PCR of C. Elegans

The total RNA of worms was extracted by Hu et al. [30], and reverse transcription was performed following the instructions of the corresponding kit. Three replicate wells were set for each sample. The *β-actin* gene was used as an internal control. The primer sequences and conditions for each gene are listed in Tables S1 and S3.

### 2.19. Data Analysis

All experiments were performed in triplicate. Statistical analyses were conducted using GraphPad Prism 9 software (GraphPad Software, San Diego, CA, USA). Data were presented as the mean ± standard deviation (SD). For multiple group comparisons, one-way or two-way ANOVA was employed, followed by an appropriate post hoc test. For comparisons between two groups, Student’s *t*-test was used. Survival data were analyzed by the Kaplan–Meier method, and the log-rank test was applied for curve comparisons. The mean lifespan was calculated from three independent experiments, with the standard error of the mean (SEM) derived from these replicates. *p* < 0.05 was considered to be statistically significant.

## 3. Results

### 3.1. Qualitative Analysis of the Components in RCAEA by LC-MS/MS

**Figure 1:**
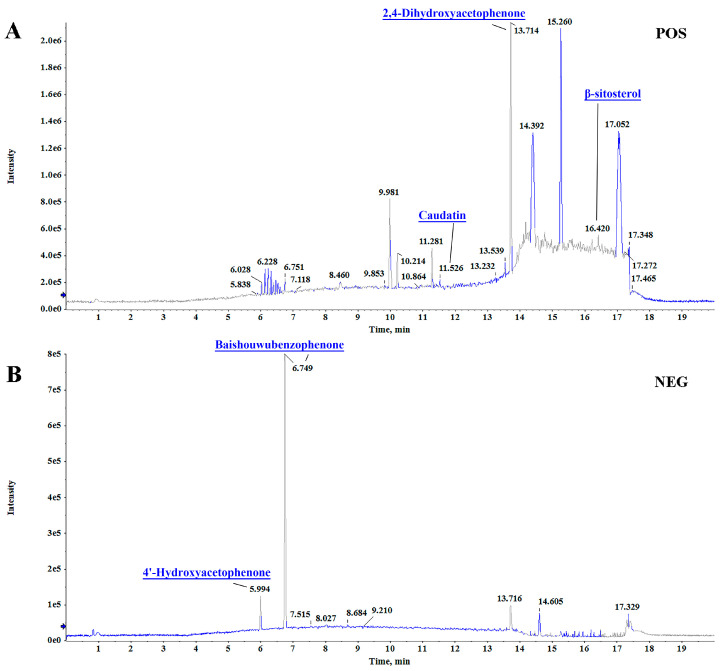
Qualitative analysis of the chemical components in RCAEA. Total ion chromatograms of RCAEA in the positive ion mode (**A**) and negative ion mode (**B**).

In total, 0.93 g of the ethyl acetate extract was obtained from the fresh roots of RCA samples (100 g). The compounds of RCAEA are shown in Figure 1. Five constituents were tentatively identified based on retention times, formulas, and error values (Table 1). The published literature provided some useful information for the identification of compound structures [16,17].

### 3.2. Predict the Anti-Aging Mechanism of RCAEA by Network Pharmacology Analysis

**Figure 2:**
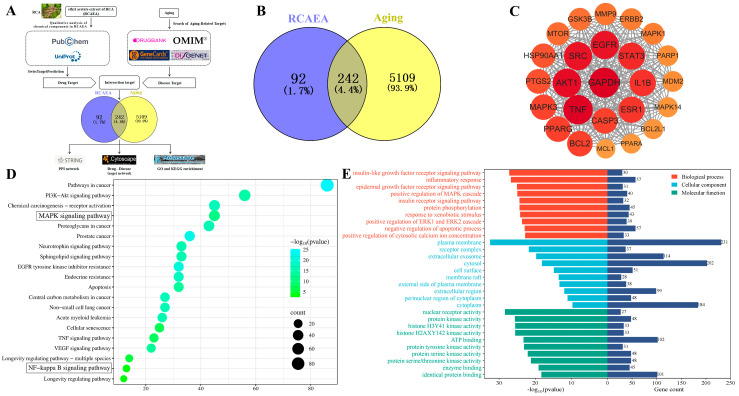
Network pharmacology revealed the key anti-aging mechanisms of RCAEA. (**A**) Process of network pharmacology analysis. (**B**) Venn diagram showing the overlap between RCAEA and aging. (**C**) PPI network of core targets. (**D**) KEGG pathway enrichment analysis of RCAEA. (**E**) GO functional enrichment analysis of RCAEA.

According to the process shown in Figure 2A, after importing the active ingredients of RCAEA into SwissTargetPrediction, 334 corresponding targets were obtained. Then, 5351 potential disease targets were identified from four disease databases. Intersection analyses revealed 242 candidate anti-aging targets of RCAEA (Figure 2B). The protein–protein interaction (PPI) of the core target interaction map showed that AKT1, SRC, EGFR, and TNF had higher node degrees (Figure 2C). Then, KEGG pathways were determined through enrichment analysis, including the regulation of the mitogen-activated protein kinase (MAPK) signaling pathway and the NF-kappa B signaling pathway (Figure 2D). GO analyses indicated that the biological process (BP) mainly involved inflammatory responses. Cellular components (CCs) mainly included the plasma membrane. Molecular functions (MFs) mainly included nuclear receptor activity (Figure 2E). This fully demonstrates that RCAEA improves aging through diverse biological mechanisms that regulate cellular and molecular functions.

### 3.3. RCAEA Suppresses M1-Type Polarization in RAW264.7 Macrophages Under Inflammatory Environment

**Figure 3:**
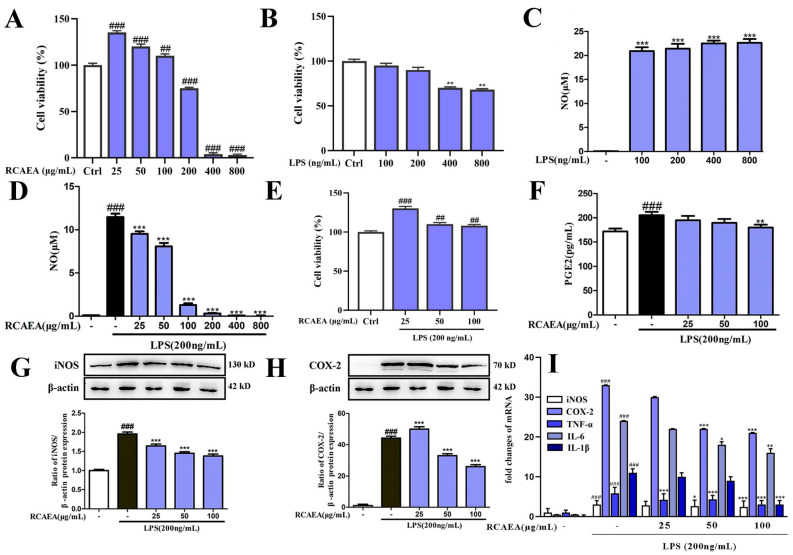
RCAEA reduces the production of inflammatory factors in LPS-induced RAW264.7 cells. (**A**) Cytotoxicity of RCAEA in RAW264.7 cells. (**B**) Cytotoxicity of LPS in RAW264.7 cells. (**C**) The release level of NO in RAW264.7 cells treated with LPS. (**D**) The release level of NO in LPS-induced RAW264.7 cells treated with RCAEA. (**E**) Cytotoxicity in LPS-induced RAW 264.7 cells treated with RCAEA. (**F**) The PGE2 level in LPS-induced RAW264.7 macrophages. (**G**,**H**) Effects of RCAEA on iNOS and COX-2 expression levels at the protein level. *β-actin* was used as the internal control for normalization. (**I**) The mRNA levels of inflammation factors. Values = mean ± SD, *n* = 3 (* *p* < 0.05, ** *p* < 0.01, and *** *p* < 0.001 compared to the LPS-treated group; ^##^ *p* < 0.01 and ^###^ *p* < 0.001 compared to the control group).

Firstly, cell viability assays confirmed that neither RCAEA (25, 50, and 100 μg/mL) nor LPS (100 and 200 ng/mL) significantly affected cell survival (Figure 3A,B). The LPS treatment (100, 200, 400, and 800 ng/mL) promoted macrophage polarization into the M1-type, characterized by increased production levels of NO (Figure 3C) [31]. In total, 200 ng/mL of LPS was finally chosen for further experiments based on cytotoxicity assessment. RCAEA samples measuring 25, 50, and 100 μg/mL notably inhibited the LPS (200 ng/mL)-induced production of NO (Figure 3D) and improved cell viability (Figure 3E). These concentrations were selected for subsequent experiments.

Excessive iNOS expression and NO production are key markers of the inflammatory response. RCAEA downregulated the expression of iNOS at both the protein and mRNA levels (Figure 3G,I). Similarly, RCAEA inhibited the production of COX-2 and PGE2 (Figure 3F,H,I). It also reduced the mRNA levels of tumor necrosis factor-alpha (TNF-α), interleukin-6 (IL-6), and IL-1β (Figure 3I). Taken together, RCAEA exhibited significant inhibitory activity against inflammation.

### 3.4. RCAEA Blocks NF-κB Pro-Inflammatory Signaling Pathway

**Figure 4:**
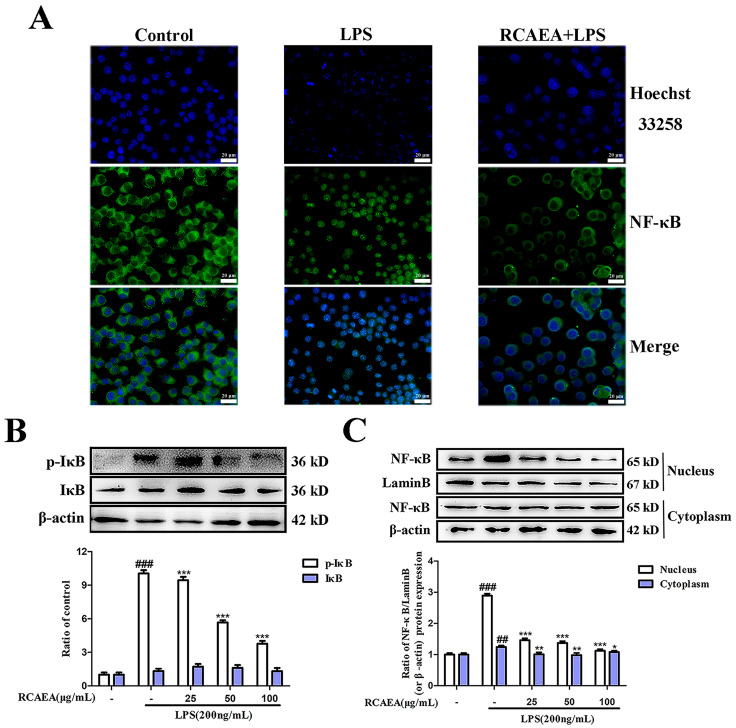
RCAEA modulated macrophage polarization and reduced the production of inflammatory factors in LPS-induced RAW264.7 cells via the NF-κB signaling pathway. (**A**) Localization images of NF-κB p65 in RAW264.7 cells. (**B**) Effects of RCAEA on LPS-induced IκBα phosphorylation. (**C**) Effects of RCAEA on LPS-induced NF-κB activation. *Lamin B* and *β-actin* were used as internal parameters for normalization. Values = mean ± SD, *n* = 3 (* *p* < 0.05, ** *p* < 0.01, and *** *p* < 0.001 compared to the LPS-treated group; ^##^ *p* < 0.01 and ^###^ *p* < 0.001 compared to the control group).

Inflammation is related to the regulation of the NF-κB signaling pathway [32]. The results suggest that NF-κB was transferred from the cytoplasm to the nucleus, and the protein expression of NF-κB was increased in the nucleus in LPS-treated RAW264.7 macrophages (Figure 4A,C). Meanwhile, the protein expression level of the phosphorylated NF-κB inhibitor α (IκBα) decreased after RCAEA treatment (Figure 4B), which reduced the nuclear transfer capacity of NF-κB and thus decreased the production of inflammatory cytokines [33]. The data suggest that RCAEA might regulate the expression of inflammatory cytokines to inhibit macrophage polarization via the NF-kB signaling pathway.

### 3.5. The Influence of RCAEA on the Biological Behaviors and Aging Process of C. elegans

**Figure 5:**
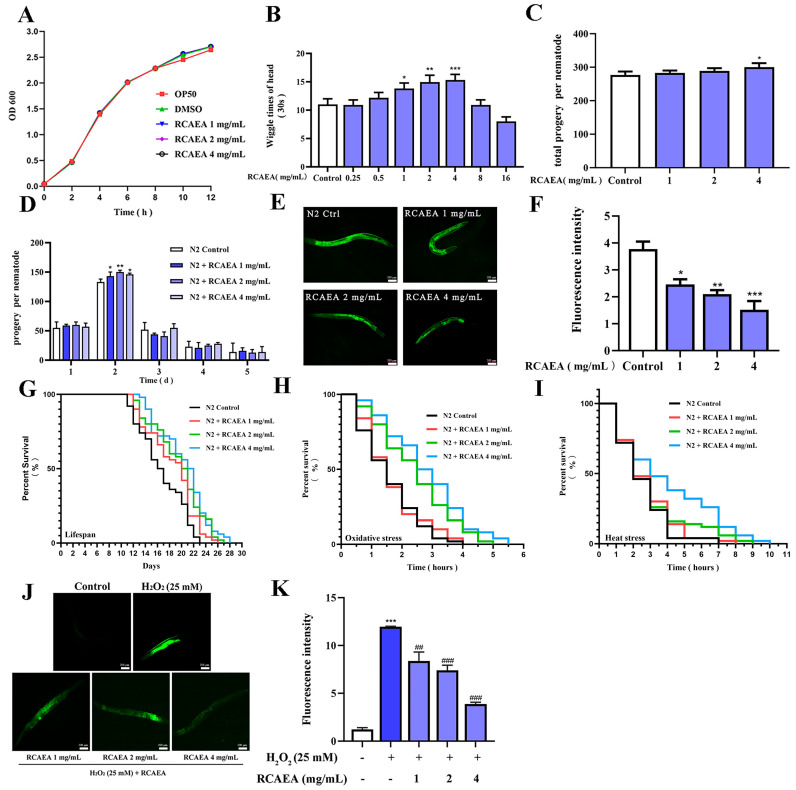
RCAEA improves biological behaviors and longevity in N2 *C. elegans*. (**A**) Effect of RCAEA on the growth rate of *E. coli* OP50. (**B**) RCAEA showed no toxicity in *C. elegans* (*n* = 30). (**C**,**D**) RCAEA showed no reproductive toxicity on N2 *C. elegans* (*n* = 5). (**E**,**F**) RCAEA reduces lipofuscin accumulation in N2 *C. elegans*. Lipofuscin accumulation was quantified using ImageJ software (*n* = 50). (**G**) RCAEA extended the lifespan of N2 *C. elegans* (*n* = 50, log-rank test). (**H**,**I**) RCAEA protected *C. elegans* from the damage induced by juglone and 35 °C heat (*n* = 50, log-rank test). (**J**,**K**) RCAEA reduced the ROS level induced by H_2_O_2_ (*n* = 50). Values = mean ± SD, *n* = 3 (* *p* < 0.05, ** *p* < 0.01, and *** *p* < 0.001 compared to the control group; ^##^ *p* < 0.01 and ^###^ *p* < 0.001 compared to the H_2_O_2_-treated group).

As shown in Figure 5A, RCAEA exhibited no anti-bacterial activity against *E. coli* OP50, confirming that subsequent effects on *C. elegans* were not due to microbial growth inhibition. As *C. elegans* age, their head-swinging frequency gradually declines until they ultimately die [34]. High doses of RCAEA (8 and 16 mg/mL) may have had toxic effects on N2 *C. elegans*, while low doses of RCAEA (0.25 and 0.5 mg/mL) had no significant influence on *C. elegans*. Therefore, 1, 2, and 4 mg/mL were selected for further analysis. RCAEA-treated (1, 2, 4 mg/mL) worms showed significantly higher head wiggle frequencies than controls (Figure 5B). This indicated that RCAEA improved the locomotor function of worms. The egg-laying number also reflected that RCAEA had no reproductive toxicity (Figure 5C,D).

The RCAEA treatment group significantly reduced lipofuscin accumulation, a quantitative biomarker of cellular aging [35]. This was evidenced by markedly weaker lipofuscin, which was 59.79% lower in the high-dose RCAEA group (4 mg/mL) compared to controls (Figure 5E,F) [36]. Juglone, a substance known to generate significant levels of superoxide radicals, and heating, which will lead to physiological changes, were observed. They all induce oxidative stress and reduce nematode viability [37,38]. RCAEA not only extended the lifespan of N2 worms (Figure 5G and Table 2) but also significantly increased the anti-stress capacity to prolong the maximum lifespan under juglone and heat stress compared to the controls (Figure 5H,I and Table S4). The excellent antioxidant capacity of RCAEA was also demonstrated by detecting ROS levels using the DCF-DA fluorescent probe, a well-established ROS-sensitive dye [39]. H_2_O_2_ could elevate ROS levels in *C. elegans* through externally induced oxidative stress [40]. Compared with the H_2_O_2_ group, RCAEA treatment at 4 mg/mL resulted in the lowest fluorescence intensity (Figure 5J,K). Based on the above experiments, it can be inferred that RCAEA may promote longevity via improving biological characteristics and anti-stress capacity in *C. elegans* [41].

### 3.6. RCAEA Prolongs the Lifespan of C. elegans via the PMK-1/SKN-1 Signaling Pathway

**Figure 6:**
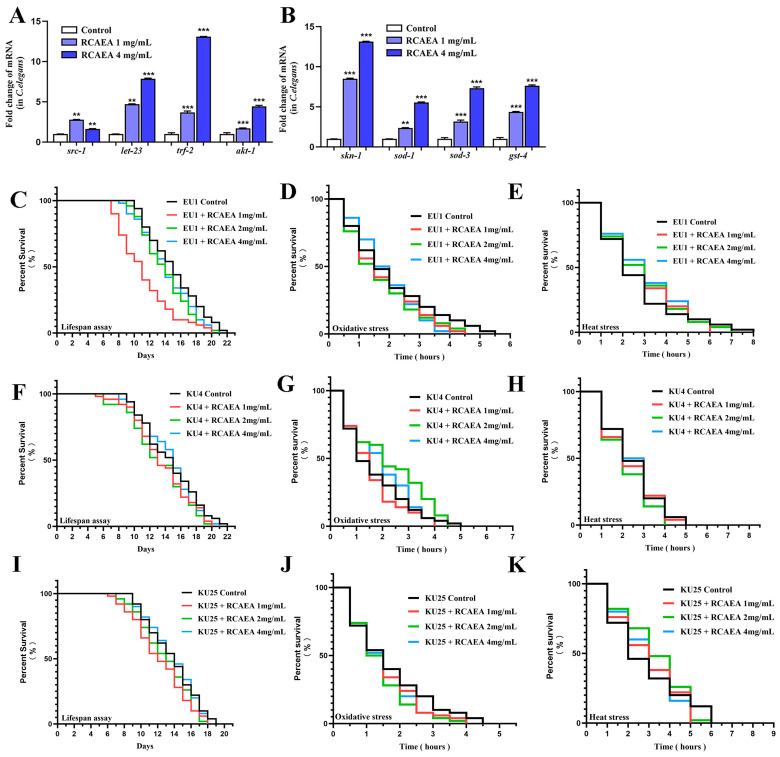
RCAEA extends the lifespan of *C. elegans* through PMK-1/SKN-1 signaling pathway. (**A**) Effects of RCAEA on anti-aging genes predicted by PPI. (**B**) RCAEA enhances the expression levels of antioxidant-related genes. (**C**–**K**) RCAEA mediates the longevity of *C. elegans* via PMK-1/SKN-1 signaling pathway (*n* = 50, log-rank test). Values = mean ± SD, *n* = 3 (** *p* < 0.01 and *** *p* < 0.001 compared to the control group).

Based on the core targets identified in the PPI analysis, we analyzed the expression of aging-associated genes, such as *src*, *let-23*, *trf-2*, and *akt-1* in N2 *C. elegans*. They all played certain roles in the aging process (Figure 6A). Notably, RCAEA treatment significantly increased the expression levels of *skn-1*-associated genes, such as *skn-1*, *sod-1*, *sod-3*, and *gst-4* (Figure 6B). Since *skn-1* is a primary regulator of the p38 MAPK/PMK-1 pathway [42], the effects of RCAEA are likely to involve the regulation of the *skn-1*-mediated *pmk-1*.

To further explore whether RCAEA enhances anti-aging abilities through the PMK-1/SKN-1 signaling pathway, we conducted stress and lifespan assays in the EU1, KU4, and KU25 mutants. As shown in Figure 6C–K, Table 3, Table 4 and Table 5, and Tables S5–S7, when *skn-1*, *sek-1*, and *pmk-1* were deficient, RCAEA did not extend the resistance to stress and lifespan under normal and stressful conditions [43]. Collectively, these findings indicate that the RCAEA may influence stress resistance and lifespan extension via the PMK-1/SKN-1 pathway in *C. elegans*.

## 4. Discussion

During aging, the underlying proinflammatory state increases and leads to inflammatory phenomena. Macrophages undergo phenotypic adaptations in response to stimuli, such as LPS, which is typically characterized by polarization into the M1 phenotype with the robust secretion of inflammatory mediators. These inflammatory mediators may undergo mutual regulation and are mediated by each other [44]. The regulation of macrophage phenotype is important for inflammation prevention [45]. Therefore, RAW264.7 macrophages were chosen as a suitable model for the in vitro experiment [46].

RAW264.7 macrophages can polarize to the M1 phenotype by LPS, as confirmed by our research on characteristic markers, such as NO, iNOS, and COX-2. iNOS catalyzes sustained, high-output NO production, while COX-2 converts arachidonic acid into its principal bioactive product. Under physiological conditions, iNOS and COX-2 are expressed at low levels. However, their expression levels are markedly upregulated in response to inflammatory factor stimuli [47,48,49]. Here, we demonstrated that RCAEA exerts significantly anti-inflammatory effects in LPS-induced RAW264.7 cells. The NF-κB signaling pathway serves as a key target for modulating inflammatory responses, and its activity directly affects iNOS and COX-2. Under the stimulation of an inflammatory environment, phosphorylated NF-κB translocates to the nucleus to bind to DNA and upregulates the expression of inflammation-related genes [50]. However, RCAEA treatment substantially reversed this process by reducing I-κBα phosphorylation, indicating that its anti-inflammatory activity is closely associated with the NF-κB pathway.

Conversely, the NF-κB pathway can be inhibited by Nrf2/SKN-1 to alleviate the inflammatory response caused by oxidative stress. Both the NF-κB and SKN-1/Nrf2 pathways require transcriptional co-activators to initiate gene expression. Under oxidative stress, JNK and p38 MAPK can activate *skn-1.* When *skn-1* is activated, its enhanced binding to co-activators may reduce the availability of these cofactors for the NF-κB pathway, thereby suppressing the transcription of pro-inflammatory cytokines. In contrast, the activation of Nrf2/SKN-1 induces the expression of antioxidant enzymes, which collectively reduce intracellular ROS levels and indirectly suppress the activation of the NF-κB pathway. Akt-1 serves as a key upstream modulator that bridges these two signaling cascades [51,52,53,54]. Therefore, we verified this theory in *C. elegans*.

*C. elegans* is widely used for studying aging, longevity, and toxicology [55]. To assess the direct influence of RCAEA on *C. elegans*, we employed two biological behaviors studied in *C. elegans* research: head wiggle and reproductive capacity [56]. Additionally, we selected several other indicators that can directly reflect the degree of aging. Lipofuscin, an oxidative by-product, serves as a classic biomarker of aging in *C. elegans*, as excessive accumulation accelerates the aging process [57]. Meanwhile, as age advances, ROS production surpasses the clearance capacity, disrupting redox balance and inducing oxidative stress, a major contributor to aging [58]. Related to these is stress resistance, a key indicator of an organism’s ability to endure external challenges. And lifespan experiments directly evaluated the effect of RCAEA on lifespan [59,60]. Our data indicated that RCAEA enhanced the survival percentage and stress resistance of *C. elegans* by improving its antioxidant capacity.

Furthermore, we detected gene expression levels of *src-1*, *let-23*, *trf-2*, and *akt-1* according to the predictions by network pharmacology, which are the direct homologs of SRC, EGFR, TRAFs, and AKT-1, respectively. *src-1* is a crucial kinase linked to NF-κB p65 and MAPKs, and it is associated with aging [61,62]. *let-23* acts upstream of the MAPK and phosphoinositide signaling pathways [63]. *let-23* activation reduces age-related pigment accumulation and influences mobility in advanced age [64]. *trf-2* is a homolog of human TRAFs. TRAF proteins interact with p38 MAPK and NF-κB, influencing cell survival and death [65]. *akt* (protein kinase B) plays a central role in the insulin signaling pathway, with impairment of this pathway extending *C. elegans’* longevity [66]. These genes are crucial in aging. RCAEA exerts an influence on these genes, suggesting that it may modulate them via the p38 MAPK/PMK-1 pathway.

Notably, the activity of *skn-1* is regulated by the p38 MAPK/PMK-1 signaling pathway. *skn-1* belongs to the NRF/CNC protein family, shares homology with NRF2, and plays an important role in *C. elegans* [67,68]. *pmk-1* activation facilitates *skn-1* nuclear translocation, enhancing cellular oxidative stress resistance by upregulating antioxidant genes such as *gst-4* [69]. *skn-1* primarily targets *sod-1* and *sod-3* in the SOD gene family, which directly regulate ROS levels [70]. Hence, the absence of *skn-1*, *sek-1*, and *pmk-1* may prevent RCAEA from extending the lifespan of these mutants or enhancing their anti-stress resistance [71]. Our speculation has been confirmed.

Previous studies have revealed that caudatin is a C_21_ steroidal glycoside derived from the root of *Cynanchum auriculatum*, which shows attenuating inflammatory effects by inhibiting PI3K/AKT and NF-κB signaling pathways [72,73]. As the most abundant phytosterol, β-Sitosterol plays a role in modulating the inflammatory response [74,75]. β-Sitosterol reduces Akt-1 phosphorylation, which in turn decreases IκBα phosphorylation and blocks the nuclear translocation of NF-κB. Meanwhile, it activates the Nrf2/SKN-1 signaling pathway to maintain redox balance [76,77,78]. 4-hydroxyacetophenone is effective against inflammation. It has also been demonstrated to be an antipigmentation reagent via inhibiting tyrosinase activity [79]. Baishouwubenzophenone has anti-functional dyspepsia effects [80]. These independent findings strongly corroborate our bioinformatic analysis, suggesting that the anti-aging effects of RCAEA are likely mediated through these precise compound–target interactions. While this cross-validation with published data significantly strengthens our hypothesis, future work will focus on the direct experimental verification of these interactions.

This study has demonstrated that RCAEA possesses potent anti-inflammatory and anti-aging properties. We discovered that RCAEA suppresses LPS-induced M1 macrophage polarization by inhibiting the NF-κB signaling pathway and extends the lifespan of *C. elegans* via regulating the PMK-1/SKN-1 pathway. These findings significantly advance our understanding of the pharmacological mechanisms of a traditional herb, positioning RCAEA as a promising multi-target agent that links the control of chronic inflammation to the promotion of longevity. This work provides a scientific foundation for the development of RCAEA or its active components into nutraceuticals or therapeutics for age-related inflammatory diseases.

Our research has certain limitations. *C. elegans* is the primary in vivo model used, which, although powerful for initial screening, necessitates validation in more complex mammalian systems. The predictive accuracy of network pharmacology may be affected by several factors, such as interspecies differences. Therefore, verifying network pharmacology predictions with experiments will help clarify the mechanism underlying the observed pharmacological effects. Furthermore, the specific compounds within RCAEA responsible for the observed effects have not been isolated and fully characterized. Future research should focus on identifying the key bioactive molecules in RCAEA and evaluating their efficacy in murine models of aging and inflammation.

In summary, our work not only elucidates a novel mechanism of action for RCAEA but also opens up new avenues for combating aging and its associated inflammatory pathologies.

## 5. Conclusions

This study assessed the impacts of RCAEA on inflammation regulation and aging in RAW264.7 cells and *C. elegans* and demonstrated that RCAEA inhibits LPS-induced M1 polarization in RAW264.7 macrophages and suppresses inflammatory responses by modulating NF-κB signaling pathways. Importantly, RCAEA enhanced stress resistance, mitigated oxidative damage, and prolonged the lifespan through the regulation of the PMK-1/SKN-1 signaling pathway, all without adverse effects on reproduction, underscoring its potential as a promising candidate for future development into a dietary supplement or a pharmaceutical agent. Overall, this study establishes the groundwork for the practical application of RCAEA.

## Figures and Tables

**Table 1:** Chemical profiling of major ingredients in RCAEA.

t_R_	Compound Name	Formula	Precursor *m*/*z*	Reference *m*/*z*	Error (ppm)	Mode
11.669	Caudatin	C_28_H_42_O_7_	491.29912	491.29999	−1.77	[M+H]^+^
16.415	Beta-Sitosterol	C_29_H_50_O	397.38257	397.38287	−0.75	[M+H]^+^
6.741	Baishouwubenzophenone	C_16_H_14_O_6_	301.0731	301.0744	4.4	[M-H]^−^
5.994	4′-Hydroxyacetophenone	C_8_H_8_O_2_	135.0456	135.0460	3.3	[M-H]^−^
13.714	2,4-Dihydroxyacetophenone	C_8_H_8_O_3_	151.0400	151.0399	−0.4	[M+H]^+^

**Table 2:** The effects of RCAEA on the lifespan of N2 *C. elegans*.

Sample	Median Lifespan/d	Max Lifespan/d	Increase in Average Lifespan/%	*p*-Value vs. Control
Control	16.33 ± 0.17	22.33 ± 0.67	-	-
1 mg/mL RCAEA	20.22 ± 0.22	26.00 ± 0.00	16.42	0.0027 **
2 mg/mL RCAEA	20.50 ± 0.29	26.33 ± 0.33	17.91	0.0029 **
4 mg/mL RCAEA	21.33 ± 0.17	27.67 ± 0.33	23.88	0.0010 ***

Data are expressed as the mean ± SEM (*n* = 3); ** *p* < 0.01, and *** *p* < 0.001.

**Table 3:** The effects of RCAEA on the lifespan of the EU1 mutant.

Sample	Median Lifespan/d	Max Lifespan/d	Increase in Average Lifespan/%	*p*-Value vs. Control
Control	14.67 ± 0.33	21.67 ± 0.33	-	-
1 mg/mL RCAEA	11.67 ± 0.33	20.33 ± 0.33	−6.15	ns
2 mg/mL RCAEA	13.67 ± 0.33	20.67 ± 0.33	−4.62	ns
4 mg/mL RCAEA	14.00 ± 0.00	20.67 ± 0.33	−4.62	ns

Data are expressed as the mean ± SEM (*n* = 3); ns = not significant.

**Table 4:** The effects of RCAEA on the lifespan of the KU4 mutant.

Sample	Median Lifespan/d	Max Lifespan/d	Increase in Average Lifespan/%	*p*-Value vs. Control
Control	14.67 ± 0.33	21.67 ± 0.33	-	-
1 mg/mL RCAEA	12.67 ± 0.33	20.33 ± 0.33	−6.15	ns
2 mg/mL RCAEA	13.00 ± 0.00	21.00 ± 0.58	−3.08	ns
4 mg/mL RCAEA	14.33 ± 0.33	20.67 ± 0.33	−4.62	ns

Data are expressed as the mean ± SEM (*n* = 3); ns = not significant.

**Table 5:** The effects of RCAEA on the lifespan of the KU25 mutant.

Sample	Median Lifespan/d	Max Lifespan/d	Increase in Average Lifespan/%	*p*-Value vs. Control
Control	13.67 ± 0.33	18.67 ± 0.33	-	-
1 mg/mL RCAEA	11.33 ± 0.33	17.67 ± 0.33	−5.36	ns
2 mg/mL RCAEA	13.00 ± 0.58	17.00 ± 0.58	−8.93	ns
4 mg/mL RCAEA	12.67 ± 0.67	17.33 ± 0.33	−7.14	ns

Data are expressed as the mean ± SEM (*n* = 3); ns = not significant.

## Data Availability

The original contributions presented in this study are included in the article/Supplementary Materials. Further inquiries can be directed to the corresponding author(s).

## Supplementary Materials

The supporting information can be downloaded at: https://www.mdpi.com/article/10.3390/cimb47110934/s1.

## Abbreviations

Akt, protein kinase B; BP, biological process; CA, *Cynanchum auriculatum* Royle ex Wight.; *C. elegans*, *Caenorhabditis elegans*; CGC, Caenorhabditis Genetics Center; CC, cellular component; COX-2, Cyclooxygenase-2; DCF-DA, 2′,7′-Dichlorofluorescein Diacetate; DMEM, Dulbecco’s Modified Eagle Medium; DNA, deoxyribonucleic acid; *E. coli* OP50, *Escherichia coli* OP50; ELISA, Enzyme-Linked Immunosorbent Assay; GO, Gene Ontology; IL-1β, interleukin-1β; IL-6, interleukin-6; KEGG, Kyoto Encyclopedia of Genes and Genomes; LPS, lipopolysaccharide; LC-MS/MS, liquid chromatography–tandem mass spectrometry; MTT, methyl thiazolyl tetrazolium; MAPK, mitogen-activated protein kinase; MF, molecular function; NGM, nematode growth medium; NP, network pharmacology; NO, nitric oxide; iNOS, inducible nitric oxide synthase; NF-κB, nuclear factor kappa-B; PPI, protein–protein interaction; PGE2, prostaglandin E2; RIPA, radio immunoprecipitation assay; RCA, *Cynanchi Auriculati Radix*; RCAEA, the ethyl acetate extract of RCA; ROS, reactive oxygen species; qRT-PCR, real-time quantitative PCR assay; NaN_3_, sodium azide; TCMSP, Traditional Chinese Medicine Systems Pharmacology; TNF-α, tumor necrosis factor-α.
