# Supplementary material for: Ethyl Acetate Extract of Cynanchi Auriculati Radix Inhibits LPS-Induced M1 Polarization of RAW264.7 Macrophages and Prolongs the Lifespan of Caenorhabditis elegans by Regulating NF-κB and PMK-1/SKN-1 Signaling Pathways
Source: Curr Issues Mol Biol. 2025 Nov 10;47(11):934. doi: 10.3390/cimb47110934 (PMC12650852; doi:10.3390/cimb47110934)
Supplement: Supplementary file 1 [file cimb-47-00934-s001.zip › cimb-3950971-supplementary.pdf]

Supporting Information for

Ethyl Acetate Extract of *Cynanchi Auriculati Radix* Inhibits LPS-Induced M1 Polarization of RAW264.7 Macrophages and Prolongs the Lifespan of *Caenorhabditis elegans* by Regulating NF-κB and PMK-1/SKN-1 Signaling Pathways

Jiawei Fan, Ya Su, Yi Xing, Kun Hu, Jie Ren * and Jia Yang *

School of Pharmacy, Changzhou University, 21 Gehu Road, Changzhou 213164, China

* Correspondence: renjie@cczu.edu.cn (J.R.); yangjia06150204@163.com (J.Y.)

Method

2.3. LC-MS/MS instrumentation and conditions

Chromatographic separation was achieved on a ExcsepTM C18 (5μm，4.6 × 250mm). The mobile phase consisted of phase A (25 mM am-monium acetate and 25 mM ammonia in water) and phase B (acetonitrile), with a flow rate of 0.3 mL/min at 40 °C. Gradient elusion was applied, 0 - 55 min，20% - 90% B；55 - 60 min，20% B, followed by re-equilibration. The data were acquired in both positive and negative modes.Mass spectrometry operating parameters were set as follows: ion source temperature, 550 °C; ion spray voltage, 5500 V in positive mode, -4500 V in nega-tive mode; curtain gas, 35 psi; gas 1, 50 psi; gas 2 50 psi. The declustering potential and collision energy were set at ± 60 V (positive: 60 V; negative: -60 V) and ± 35 eV (positive: 35 eV; negative: -35 eV) with a spread of 15 eV, respectively. The data was analyzed by Peakview SoftwareTM v. 1.2 (AB SCIEX).

2.6. ELISA

RAW264.7 cells were treated with RCAEA (25, 50, 100 μg/mL) or 200 ng/mL LPS for 24 h. After collecting the cell supernatant, we according to the ELISA kits, the standard solution was added to the standard wells, then the diluted cell supernatant was added into the sample well to be tested and incubated them at 37 °C for 30 minutes in a cell incubator. Next, dilute the concentrated washing solution 30 times with deuterium depleted water and add the washing solution to the top of each well. Add 50 μL of HRP-conjugate reagent to all the wells (except the blank well), continue to culture, and repeat the washing process. Finally, we added chromogenic solution A and chromogenic solution B together and incubated the mixture at 37 °C in the dark. The levels of prostaglandin E2 (PGE2) and interleukin-1β (IL-1β) were detected.

2.9. Immunofluorescence

RAW264.7 cells were cultured in 12-well plates and subjected to the indicated

treatments for 24 h. RAW264.7 cells were fixed with 4% paraformaldehyde at room temperature for 25 min, permeabilized with 0.1% Triton X-100 for 20 min and blocked with 1% BSA for 30 min, then incubated with primary antibody (1:100) at 4 °C overnight. Fluorescein isothiocyanate (FITC) secondary antibody was labeled to detect the localization of NF-κB was performed using 10 μg/mL Hoechst 33258 staining solution (500 μl/well) for 5 min, followed by fluorescence microscopic analysis (FSX100, OLYMPUS, Japan).

2.11. Bacterial Growth Rates

A total of 400 μL of *E. coli* OP50 and 40 mL of LB broth medium were added to the centrifuge tubes. These tubes were then placed on a thermoshaker and shaken at 37 °C for 12 h. After that, different concentrations of RCAEA (1, 2 and 4mg/mL) were added, which were shake at 37 °C. Absorbance was measured every 2 hours at 595 nm using a microplate reader for up to 8 h (Thermo Fisher, Waltham).

2.16. Measurement of reactive oxygen species (ROS)

C. elegans were cultured as described in Section 2.13 for 3 days. These worms were exposed to H_2O_2 for 4 hours. After stress induction, these worms were incubated in the dark with 0.5 mM 2′,7′-Dichlorofluorescein Diacetate (DCF-DA) for 1 hour at room temperature. Subsequently, these worms were washed twice with M9 buffer (4000r/min for 2 minutes each time) and immobilized with sodium azide (NaN_3). The fluorescence was measured using a fluorescence microplate reader at excitation/emission wavelengths of 485/530 nm, and relative ROS levels were quantified based on fluorescence intensity using ImageJ software.

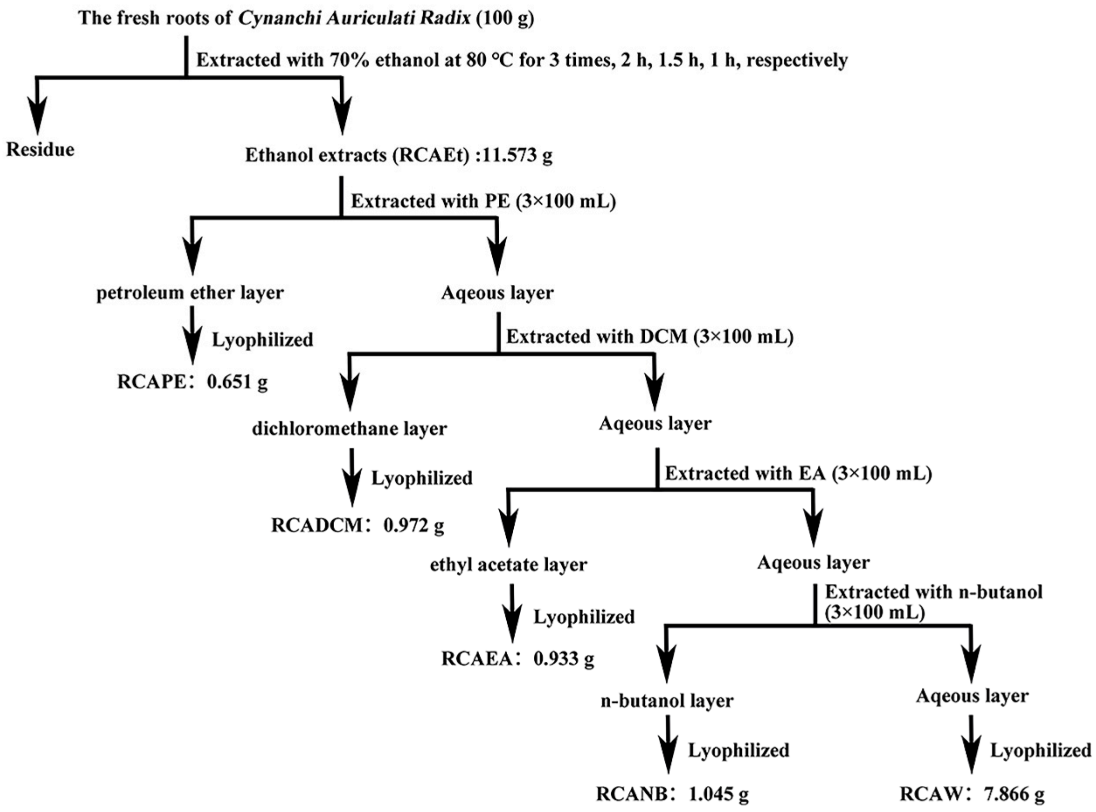

Figure S1 The preparation for RCAEA.

Table S1 The experiment condition for different genes in qRT-PCR.

Gene	Annealing/Extension
skn-1	57 ℃×30 s
sod-1	60 ℃×30 s
sod-3	60 ℃×30 s
gst-4	57 ℃×30 s
let-23	57 ℃×30 s
trf-2	55 ℃×30 s
akt-1	60 ℃×30 s
src-1	53 ℃×30 s
IL-1β	57 ℃×30 s
TNF-α	57 ℃×30 s
iNOS	57 ℃×30 s
COX-2	55 ℃×30 s
IL-6	53 ℃×30 s
β-actin	55 ℃×30 s

The PCR amplification program was as follows: initial denaturation was performed at 95 °C for 2-3 min, followed by 95 °C for 10-15 s. Annealing/extension for 30 s for 40 cycles according to mentioned temperature. Melted at 95 °C for 15 s and 65 °C for 1 min, and then the temperature increased at rate of 0.2-0.3 °C every 15 s, ending at 95 °C for 15 s.

Table S2 The Primer sequences for the inflammation-related genes of RAW264.7 cells.

Gene	Forward (5′−3′)	Reverse (5′−3′)
IL-1β	TGCCACCTTTTGACAGTGATG	TTCTTGTGACCCTGAGCGAC
TNF-α	ATAGCAGGTCTATGTGCGGC	ACAAATATTCGCCTCTGGCCT
iNOS	ACTTGTCCGCTCCTCAACAG	TCGAAAATCAGGGATGCGGC
COX-2	GGGTTCTTGTCCGATGGTGT	GGCAATCCGCAGTTGAAACA
IL-6	CACGGCCTTCCCTACTTCAC	TGCAAGTGCATCATCGTTGT
β-actin	GCTCCTCCTGAGCGCAAG	CATCTGCTGGAAGGTGGACA

Table S3 The Primer sequences for different genes of *C. elegans*.

Gene	Forward (5′−3′)	Reverse (5′−3′)
skn-1	TCCACCAGCATCTCCATTCG	CTCCATAGCACATCAATCAAGTCG
sod-1	GAGTCGGAGACAAGGCAGAAGAG	AGCAGCGAGAGCAATGACACC
sod-3	CGAGCTCGAACCTGTAATCAGCCATG	GGGGTACCGCTGATATTCTTCCAGTTG
gst-4	TCCGTCAATTCACTTCTTCCG	CACCATGAGTCCAATGATTGCA
let-23	TGTGGTGAGAGAATGGAACCT	TAAACACGACGACACCCTCG
trf-2	TTCGCTGCTGATGGTGACAT	CGACCATCTCGGACTGACAC
akt-1	CCAGGAATTGCTGATCGTATGCAGAA	TGGAGAGGGAAGCGAGGATAGA
src-1	GAATTACGCGCCACCAAGTG	AGGGGAAACAAAAACGAGGA

Table S4. Effects of RCAEA on the survival of the N2 *C. elegans* under oxidative and heat stress.

Treatment	Sample	Median Lifespan/h	Max Lifespan/h	Increase in average lifespan/%	*p*-Value vs. Control
Heat stress	Control	2.00±0.00	6.33±0.33	-	-
Heat stress	1 mg/mL RCAEA	2.33±0.33	8.33±0.33	31.58	0.0066 **
Heat stress	2 mg/mL RCAEA	2.67±0.33	8.67±0.33	36.84	0.0039 **
Heat stress	4 mg/mL RCAEA	3.00±0.00	10.67±0.33	68.42	0.0004 ***
Oxidative stress	Control	1.33±0.17	3.67±0.17	-	-
Oxidative stress	1 mg/mL RCAEA	1.67±0.17	4.33±0.17	18.18	0.0237 *
Oxidative stress	2 mg/mL RCAEA	2.33±0.17	5.17±0.17	40.91	0.0016 **
Oxidative stress	4 mg/mL RCAEA	2.67±0.08	5.50±0.00	50.00	0.0002 ***

Data are expressed as the mean ± SEM (n = 3); * $p < 0.05$, ** $p < 0.01$, *** $p < 0.001$, ns = not significant.

Table S5. Effects of RCAEA on the survival of the EU1 *C. elegans* under oxidative and heat stress.

Treatment	Sample	Median Lifespan/h	Max Lifespan/h	Increase in average lifespan/%	*p*-Value vs. Control
Heat stress	Control	2.33±0.33	7.67±0.33	-	-
Heat stress	1 mg/mL RCAEA	2.33±0.33	6.67±0.33	-13.04	ns
Heat stress	2 mg/mL RCAEA	3.00±0.00	7.00±0.00	-8.70	ns
Heat stress	4 mg/mL RCAEA	2.67±0.33	6.67±0.33	-13.04	ns
Oxidative stress	Control	1.33±0.17	4.67±0.44	-	-
Oxidative stress	1 mg/mL RCAEA	1.67±0.17	4.17±0.17	-10.71	ns
Oxidative stress	2 mg/mL RCAEA	1.50±0.29	4.00±0.29	-14.29	ns
Oxidative stress	4 mg/mL RCAEA	1.92±0.08	3.83±0.44	-17.86	ns

Data are expressed as the mean ± SEM (n = 3);-ns = not significant.

Table S6. Effects of RCAEA on the survival of the KU4 *C. elegans* under oxidative and heat stress.

Treatment	Sample	Median Lifespan/h	Max Lifespan/h	Increase in average lifespan/%	*p*-Value vs. Control
Heat stress	Control	2.00±0.00	5.33±0.33	-	-
Heat stress	1 mg/mL RCAEA	2.17±0.17	5.00±0.00	-6.25	ns
Heat stress	2 mg/mL RCAEA	1.83±0.17	4.67±0.33	-12.50	ns
Heat stress	4 mg/mL RCAEA	2.33±0.17	4.67±0.33	-12.50	ns
Oxidative stress	Control	1.33±0.17	5.33±0.17	-	-
Oxidative stress	1 mg/mL RCAEA	1.33±0.17	4.33±0.17	-18.75	ns
Oxidative stress	2 mg/mL RCAEA	1.83±0.17	4.67±0.33	-12.50	ns
Oxidative stress	4 mg/mL RCAEA	1.83±0.17	4.67±0.33	-15.63	ns

Data are expressed as the mean ± SEM (n = 3); ns = not significant.

Table S7. Effects of RCAEA on the survival of the KU25 *C. elegans* under oxidative and heat stress.

Treatment	Sample	Median Lifespan/h	Max Lifespan/h	Increase in average lifespan/%	*p*-Value vs Control
Heat stress	Control	2.00±0.00	5.33±0.33	-	-
Heat stress	1 mg/mL RCAEA	2.17±0.44	4.67±0.33	-12.50	ns
Heat stress	2 mg/mL RCAEA	2.67±0.33	5.67±0.33	-6.25	ns
Heat stress	4 mg/mL RCAEA	2.83±0.17	5.33±0.33	0.00	ns
Oxidative stress	Control	1.83±0.17	4.50±0.29	-	-
Oxidative stress	1 mg/mL RCAEA	1.67±0.17	4.33±0.17	-3.70	ns
Oxidative stress	2 mg/mL RCAEA	1.58±0.22	3.67±0.33	-18.52	ns
Oxidative stress	4 mg/mL RCAEA	1.67±0.44	4.67±0.33	3.70	ns

Data are expressed as the mean ± SEM (n = 3); ns = not significant.
